# Skeletal muscle index together with body mass index is associated with secondary osteoporosis in patients with rheumatoid arthritis

**DOI:** 10.1186/s40001-024-01665-2

**Published:** 2024-01-20

**Authors:** Yi-ran Chu, Yue-chen Xu, Ling-li Ma, Jian-xiong Wang, He-xiang Zong, Wan-qiu Tong, Xi-le Wang, Xu Zhao, Sheng-qian Xu

**Affiliations:** 1 Department of Rheumatology & Immunology, the First Affiliated Hospital of Anhui Medical University, No. 218, Ji-Xi Road, Hefei, 230022 Anhui China; 2 Department of Radiotherapy, the First Affiliated Hospital of Anhui Medical University, No. 218, Ji-Xi Road, Hefei, 230022 Anhui China

**Keywords:** Rheumatoid arthritis, Sarcopenia, Osteoporosis, Body composition

## Abstract

**Objective:**

The objective of this study was to explore the associations of body mass index (BMI), fat mass index (FMI), skeletal mass index (SMI) and secondary osteoporosis (OP) in patients with rheumatoid arthritis (RA).

**Methods:**

The bone mineral density (BMD) at sites of the femur neck (Neck), total hip (Hip) and lumbar vertebrae 1–4 (L1-4) was measured by dual-energy X-ray absorptiometry. The skeletal muscle index, body fat percentage and mineral content were measured by biological electrical impedance for calculating BMI, FMI and SMI.

**Results:**

A total of 433 patient with RA and 158 healthy controls were enrolled. The BMDs at each site of the RA patients were lower compared with those of the healthy controls (p < 0.0001), and the prevalence of OP (36.1%, 160/443) and sarcopenia (65.2%, 288/443) in the RA patients were higher than those in the controls (12.7%, 20/158, p < 0.0001; 9.0%, 14/156, p < 0.0001). Significant differences in the BMD, FMI, SMI, mineral content, body fat percentage and skeletal muscle mass were found among the RA patients in the different BMI groups (p < 0.05). In RA patients with BMI < 18.5 kg/m^2^, the prevalence of OP in the RA patients with sarcopenia was similar to that in those without sarcopenia (44.4% vs. 66. 7%, χ^2^ = 0. 574, p = 0.449). In the RA patients with a normal BMI or who were overweight or obese, prevalence of OP in the RA patients with sarcopenia was significantly higher than that in the RA patients without sarcopenia (42.8% vs. 21.7%, χ^2^ = 10.951, p = 0.001; 61.1% vs. 13.0%, χ^2^ = 26.270, p < 0.0001). In the RA patients without sarcopenia, the prevalence of OP in the RA patients in the different BMI groups was different (p = 0.039). In the RA patients with sarcopenia, there was no significant difference in the prevalence of OP among the RA patients in the different BMI groups (p = 0. 128). The linear correlation analysis showed that the SMI in RA patients was positively correlated with the BMD of each site measured and BMI and FMI (p < 0.0001). However, there was a negative linear correlation between SMI and disease duration (p = 0.048). The logistic regression analysis found that SMI (OR = 0.569, p = 0.002, 95% CI 0.399–0.810), BMI (OR = 0.884, p = 0.01, 95% CI 0.805–0.971) and gender (1 = female, 2 = male) (OR = 0.097, p < 0.0001, 95% CI 0.040–0.236) were protective factors for OP in RA, while age (OR = 1.098, p < 0.0001, 95% CI 1.071–1.125) was the risk factor.

**Conclusion:**

BMI and SMI are associated with the occurrence of OP in RA patients, and both SMI and BMI are important protective factors for OP secondary to RA.

## Key points

1. Compared with normal subjects, RA patients had lower BMD and higher prevalence of OP.
2. When BMI was fixed, the prevalence of OP in RA patients with sarcopenia was significantly higher than that in RA patients without sarcopenia. While when SMI was fixed, there was no significant difference in the prevalence of OP in RA patients with different BMI groups.
3. Both BMI and SMI are important protective factors for OP secondary to RA.

## Introduction

Rheumatoid arthritis (RA) is a systemic inflammatory autoimmune disease of unknown etiology, with symmetrical polyarthritis as the main clinical manifestation and mainly involving facet joints, and it is one of the most common diseases leading to joint deformity and disability [1, 2]. Chronic synovitis and persistent bone erosion are the main features of RA, which can lead to joint structural destruction, local or systemic bone loss and osteoporosis [2–4]. RA can also involve tendons, ligaments and other connective tissues near joints, eventually leading to joint dysfunction, deformity and disability [5]. Previous evidence has shown that RA patients have a higher probability of developing osteoporosis [6–8].

Body mass index (BMI) is calculated by taking a person's weight, in kilograms, divided by their height, in meters squared, and it is commonly used to classify a person as underweight, normal weight, overweight, or obese. Studies have reported that high BMI is a protective factor for osteoporosis, and BMI is positively correlated with bone mineral density [9, 10]. This has led to the conclusion that obesity protects against osteoporosis and fractures [11]. Yet this conclusion may be too partial. In fact, both fat and lean mass are increased in obese humans, and the effect of BMI on bone mineral density is determined by the interaction between bone tissue muscle tissue and adipose tissue, and individuals with similar BMIs may have diverse body compositions. Muscle tissue has multiple functions, including maintaining body posture and collaborating with the skeletal system to support movement, and muscle loss may increase the risk of fall, osteoporosis and fracture [12].

Skeletal mass index (SMI) is calculated by dividing the skeletal muscle content of the extremities by the square of the height, and it is used to represent the muscle content in the human body. Fat mass index (FMI) is calculated by dividing the fat mass by the square of the height, and it is used to express the level of body fat content. Present studies have focused on the correlation between BMI and osteoporosis in RA patients but ignore the role of SMI and FMI in RA secondary osteoporosis. In this paper, we added a correlation study of SMI, FMI and RA secondary osteoporosis, and explores the relationship between BMI, SMI and FMI.

## Materials and methods

### Study population

RA patients hospitalized at the Department of Rheumatology and Immunology of the First Affiliated Hospital of Anhui Medical University from 1 June 2017 to 31 December 2021 were enrolled in our study. The diagnosis of RA also fulfilled the 1987 American College of Rheumatology (ACR) diagnostic criteria for RA classification [13] or the 2010 ACR/European League Against Rheumatism (EULAR) criteria for RA. Age- and gender-matched healthy people who visited the physical examination Center of the First Affiliated Hospital of Anhui Medical University were selected as the control group, and we matched the control group with the RA group by a ratio of one to three. Patients with endocrine metabolic diseases (such as thyroid functions of hyperthyroidism and hypothyroidism), acute or chronic infectious diseases, and severe liver and kidney disease or patients with a primary blood disease were excluded. Patients who used anticonvulsants, anticoagulants, estrogens, androgens, antidepressants and other psychotropic drugs were also excluded. Other exclusion criteria included alcoholics, smokers, pregnant or lactating patients and patients with non-RA inflammatory arthritis, major trauma, infectious and inflammatory diseases, and other decompensated diseases. Exclusion criteria were applied to both RA and control groups, and none of the RA patients had used biological agents. This study was approved by the Ethics Committee of Anhui Medical University, and all participants signed written informed consent (approval number: 20121090).

### Data collection

The general characteristics of all of the participants were recorded in our study, including age, sex, height, weight, usage of glucocorticoids (GCs) and disease duration. Serum 25 (OH)D levels were measured in all subjects. All RA patients were assessed with the 28 joint disease activity score (DAS28-ESR) by the same senior rheumatologist according to the standard formula and clinical disease activity indicators. A healthy assessment questionnaire (HAQ) was used for assessing the whole functional status. The body composition indexes were measured by direct segmental multifrequency bioelectrical impedance testing using a body composition analyzer (Inbody 720: Biospace Co., Ltd., Seoul, Korea). The BMDs (g/cm^2^) of the femoral neck (Neck), total Hip (Hip) and lumbar spine 1–4 (L1-4) were measured by dual-energy X-ray absorptiometry (Lunar Prodigy DF + 310,504, GE Healthcare, USA). All patients completed the clinical interview on the day of admission, including the basic information of the patient, HAQ, and other questionnaires. Clinical examinations including blood tests, DXA, body composition, and DAS28 were completed on the second day of admission.

### BMI grouping

According to the BMI grouping criteria [14], BMI < 18.5 kg/m^2^ was defined as underweight; BMI ≥ 18.5 kg/m^2^ and < 24.0 kg/m2 was the normal level; BMI ≥ 24.0 kg/m^2^ and < 28.0 kg/m^2^ was overweight; and BMI ≥ 28.0 kg/m^2^ was obesity.

### Diagnostic criteria for osteoporosis and sarcopenia

According to the WHO diagnostic criteria for osteoporosis [15], BMD was defined as normal if it was within 1 standard deviation below the peak value of healthy people of the same gender and race. A decrease of 1–2.5 standard deviations was defined as osteopenia. A decrease of 2.5 standard deviations or more was considered to be osteoporosis. Fractures that occurred without trauma or minor trauma and fragility fractures that occurred with decreased bone strength could also be diagnosed as osteoporosis. Severe osteoporosis was defined as one or more fragility fractures accompanied by a bone mineral density reduction meeting the diagnostic criteria for osteoporosis.

SMI was derived by dividing the appendiceal skeletal muscle mass (kg) by the square of height (m). Sarcopenia was diagnosed in accordance with the Asian Working Group for Sarcopenia criteria and was defined as SMI < 5.7 kg/m^2^ in women and < 7.0 kg/m^2^ in men, corresponding to a value 2 standard deviations below the mean of the young reference group [16].

### Statistical analysis

Continuous data with normal distribution and skewed distribution are represented as the mean ± standard error and the median (interquantile range), respectively, and categorical data are displayed as absolute value (percentage of sub/group). The between-group comparison of continuous variables was conducted by the t-test or Wilcoxson rank test according to the distribution, and the between-group comparison of the categorical variables used the chi-square test. The Pearson correlation test was used to evaluate the correlation between SMI and BMD, BMI, FMI, body fat percentage, disease duration and DAS28-ESR, and the correlation analysis was expressed as a correlation coefficient r. A multiple logistic regression analysis was used for the multivariate analysis. Statistical analyses were performed using SPSS version 17.0 and a p-value of < 0. 05 was considered statistically significant.

## Results

### Comparison between the RA patients and normal groups

**Fig. 1:**
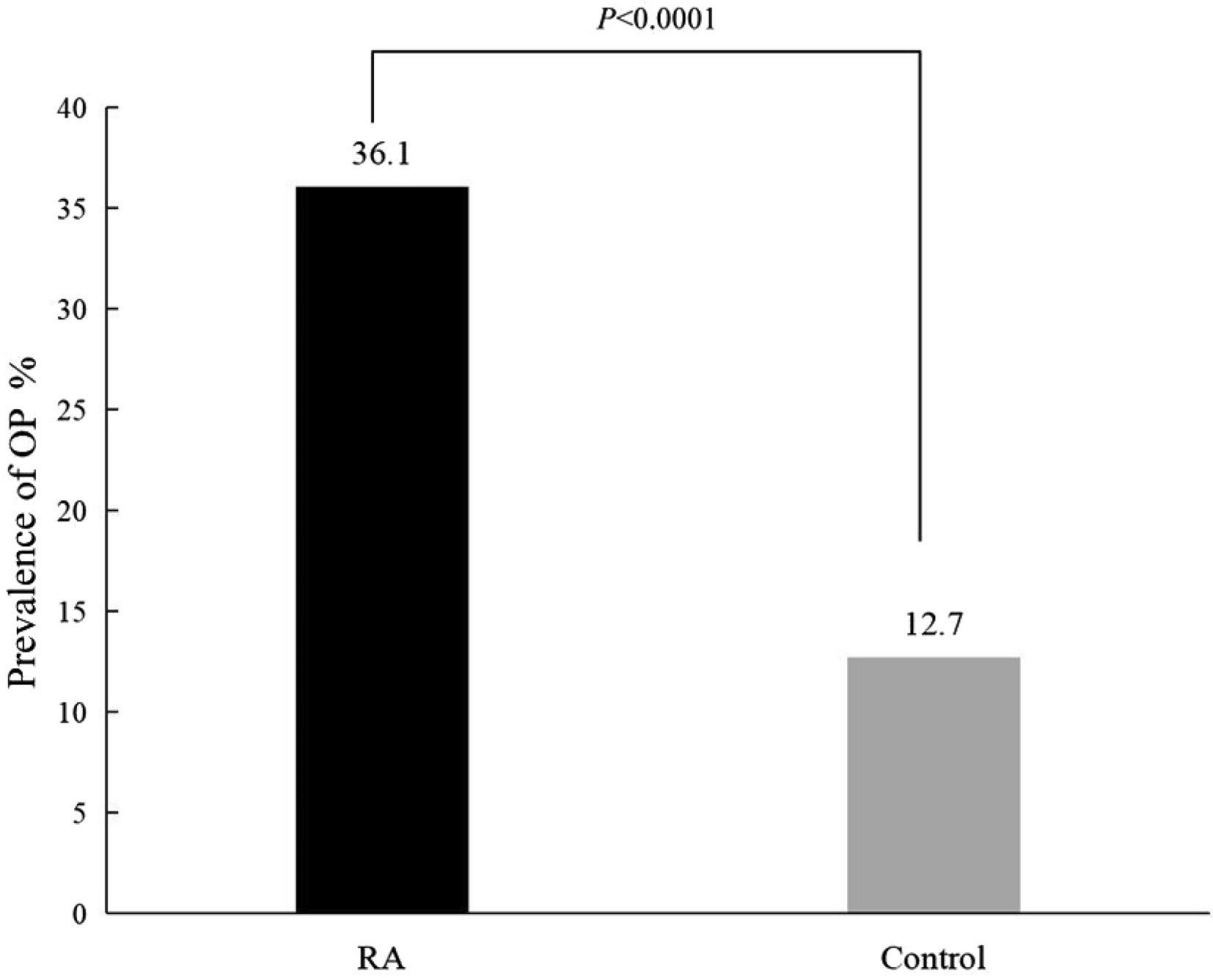
Comparison of OP prevalence between RA and control group

The study sample include 443 RA patients and 158 controls. There were no significant differences in age (z = 1.081, p = 0.280), weight (z = 0.473, p = 0.636), height (z = 1.118, p = 0.264), BMI (t = 0.446, p = 0.656), gender composition (χ^2^ = 0.175, p = 0.676) and proportion of postmenopausal women (χ^2^ = 0.685, p = 0.450) between the two groups (Table 1).The BMDs of Neck, Hip, L1, L2, L3, L4 and L1-4 in the RA patients were significantly lower than those in the healthy controls (p < 0.001) (Table 2). The prevalence of osteoporosis in the RA patients was 36.1% (160/443), which was significantly higher than that in the control group at 12.7% (20/158) (χ^2^ = 30.550, p < 0.0001) (Fig. 1). The prevalence of sarcopenia in the RA patients was 65.2% (288/443), which was significantly higher than that in the control group at 9.0% (14/156) (χ^2^ = 145.605, p < 0.0001).

**Table 1:** Characteristics between RA patients and controls

Indicators	RA (n = 443)	Control (n = 158)	t/z/*χ*^2^	*p*
Age (year)	57.00 (50.00–68.00)	58.00 (47.00–66.00)	1.081	0.280
Female, n (%)	352 (79.5%)	128 (81.0%)	0.175	0.676
Weight (kg)	55.00 (50.00–62.00)	55.05 (49.95–63.30)	0.473	0.636
Height (cm)	160.00 (155.00–165.00)	160.00 (156.00–166.00)	1.118	0.264
BMI (kg/m^2^)	21.83 ± 3.48	21.96 ± 2.86	0.446	0.656
Postmenopausal Women, n (%)	269 (60.7%)	90 (57.0%)	0.685	0.450
RA with hypertension, n (%)	103 (23.3%)	–	–	–
RA with diabetes, n (%)	32 (7.2%)	–	–	–

**Table 2:** Comparison of BMD and 25 (OH)D between RA and control group

Indicators	RA (n = 443)	Control (n = 158)	t/z	*p*
Neck BMD (kg/m^2^)	0.76 (0.66–0.87)	0.92 (0.81–1.03)	9.375	< 0.0001
Hip BMD (kg/m^2^)	0.81 ± 0.14	0.97 ± 0.15	12.160	< 0.0001
L1 BMD (kg/m^2^)	0.89 ± 0.17	1.04 ± 0.15	10.353	< 0.0001
L2 BMD (kg/m^2^)	0.93 ± 0.18	1.07 ± 0.19	8.183	< 0.0001
L3 BMD (kg/m^2^)	1.01 ± 0.19	1.14 ± 0.19	7.313	< 0.0001
L4 BMD (kg/m^2^)	1.03 ± 0.19	1.14 ± 0.18	6.488	< 0.0001
L1-4 BMD (kg/m^2^)	0.99 ± 0.18	1.12 ± 0.18	7.579	< 0.0001
25 (OH)D (ng/ml)	18.10 (12.80–23.80)	21.82 (17.95–25.81)	4.694	< 0.0001

### Comparison in different groups

**Fig. 2:**
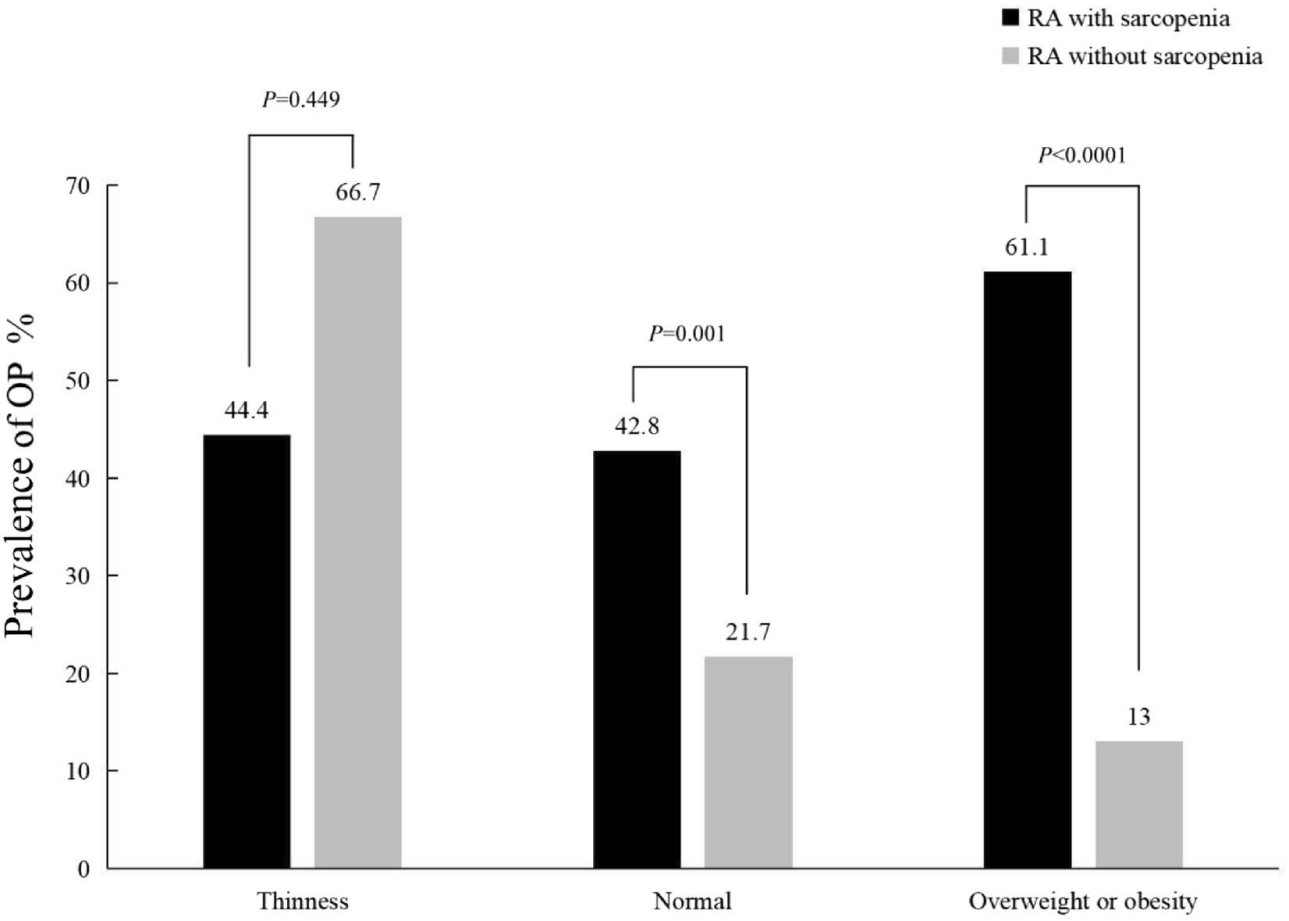
Comparison of OP prevalence by SMI in different weight groups

According to the BMI, the RA patients were divided into three groups: underweight, normal and overweight or obesity. There were significant differences in BMD, SMI, FMI, mineral content, body fat percentage and skeletal muscle content among the three groups (p < 0.05–0.0001) (Table 3).In thin RA patients, the prevalence of osteoporosis in the sarcopenia group was similar to that in the nonsarcopenia group (44.4% vs. 66.7%, χ^2^ = 0. 574, p = 0.449). In the normal BMI, overweight or obese groups, the prevalence of osteoporosis in the sarcopenia group was significantly higher than that in the nonsarcopenia group (42.8% vs. 21.7%, χ^2^ = 10.951, p = 0.001; 61.1% vs. 13.0%, χ^2^ = 26.270, p < 0.0001) (Fig. 2). In the RA patients without sarcopenia, the prevalence of osteoporosis in the RA patients in the different BMI groups were different (66.7%, 21.7% and 13.0%, p = 0.039), while in the RA patients with sarcopenia, the prevalence of osteoporosis in the RA patients in the different BMI groups were not significantly different (44.4%, 42.8% and 61.1%, p = 0.128).

**Table 3:** Comparison of different indicators in RA patients by BMI groups

Indicators	Thinness (n = 75)	Normal (n = 263)	Overweight or obesity (n = 105)	F/z	*p*
Neck BMD (g/m^2^)	0.71 (0.66–0.81)	0.77 (0.66–0.88)	0.80 (0.70–0.92)	8.472	0.014
Hip BMD (g/m^2^)	0.76 ± 0.13	0.81 ± 0.14	0.85 ± 0.15	8.921	< 0.0001
L1 BMD (g/m^2^)	0.81 (0.70–0.91)	0.89 (0.78–1.02)	0.91 (0.81–1.02)	16.246	< 0.0001
L2 BMD (g/m^2^)	0.86 ± 0.15	0.95 ± 0.18	0.96 ± 0.17	8.863	< 0.0001
L3 BMD (g/m^2^)	0.92 ± 0.16	1.02 ± 0.19	1.05 ± 0.18	10.987	< 0.0001
L4 BMD (g/m^2^)	0.92 ± 0.15	1.04 ± 0.19	1.07 ± 0.17	15.191	< 0.0001
L1-4 BMD (g/m^2^)	0.90 (0.79–0.97)	0.97 (0.86–1.10)	1.00 (0.90–1.13)	21.754	< 0.0001
Mineral content (g)	1.94 (1.78–2.70)	2.13 (1.96–2.36)	2.23 (2.02–2.41)	48.531	< 0.0001
Skeletal muscle mass (g)	30.50 (28.00–33.80)	33.80 (30.90–37.90)	35.80 (32.55–39.65)	41.685	< 0.0001
Body fat percentage (%)	22.97 ± 7.26	31.55 ± 7.39	39.49 ± 7.05	113.561	< 0.0001
SMI (g/m^2^)	4.86 (4.26–5.38)	5.51 (5.06–6.12)	6.10 (5.59–6.76)	77.412	< 0.0001
FMI (g/m^2^)	3.91 (2.88–4.97)	6.98 (5.58–7.98)	10.18 (8.95–11.99)	240.588	< 0.0001

According to dosage of glucocorticoids, RA patients were divided into three groups: none, ≤ 10 mg/d, > 10 mg/d. There were no significant differences in BMD, FMI, BMI and body fat percentage among the three groups (p > 0.05). And there were significant differences in skeletal muscle mass, mineral content and SMI among the three groups (p < 0. 05) (Table 4).

**Table 4:** Comparison of different indicators in RA patients by dosage of glucocorticoids groups

Indicators	None (N = 195)	≤ 10 mg/d (N = 206)	> 10 mg/d (N = 42)	F/z	*p*
Neck BMD (g/m^2^)	0.78 (0.67–0.88)	0.75 (0.65–0.86)	0.74 (0.68–0.87)	1.254	0.534
Hip BMD (g/m^2^)	0.83 ± 0.15	0.80 ± 0.14	0.81 ± 0.12	2.412	0.091
L1 BMD (g/m^2^)	0.90 ± 0.16	0.89 ± 0.18	0.87 ± 0.18	0.937	0.393
L2 BMD (g/m^2^)	0.93 (0.81–1.07)	0.91 (0.81–1.05)	0.94 (0.80–1.01)	1.058	0.589
L3 BMD (g/m^2^)	1.02 ± 0.19	1.01 ± 0.19	1.00 ± 0.17	0.234	0.792
L4 BMD (g/m^2^)	1.02 (0.91–1.17)	0.99 (0.89–1.15)	1.04 (0.92–1.16)	1.716	0.424
L1-4 BMD (g/m^2^)	0.98 ± 0.17	0.96 ± 0.17	0.96 ± 0.15	0.873	0.418
Mineral content (g)	2.18 (1.94–2.39)	2.07 (1.92–2.28)	2.12 (1.96–2.35)	6.512	0.039
Skeletal muscle mass (g)	35.10 (31.00–39.00)	33.10 (30.08–36.70)	33.90 (31.03–36.65)	11.154	0.004
Body fat percentage (%)	31.17 ± 8.42	32.66 ± 9.15	32.37 ± 10.24	1.427	0.241
SMI (g/m^2^)	5.72 (5.12–6.41)	5.41 (4.82–6.07)	5.56 (4.82–6.13)	11.952	0.003
FMI (g/m^2^)	7.17 (5.15–8.63)	7.03 (5.16–9.03)	7.32 (5.15–9.82)	0.724	0.696
BMI (g/m^2^)	22.19 (20.03–23.88)	21.65 (19.11–23.68)	22.05 (19.02–24.14)	1.847	0.397

### The linear correlation and logistic regression analysis

**Fig. 3:**
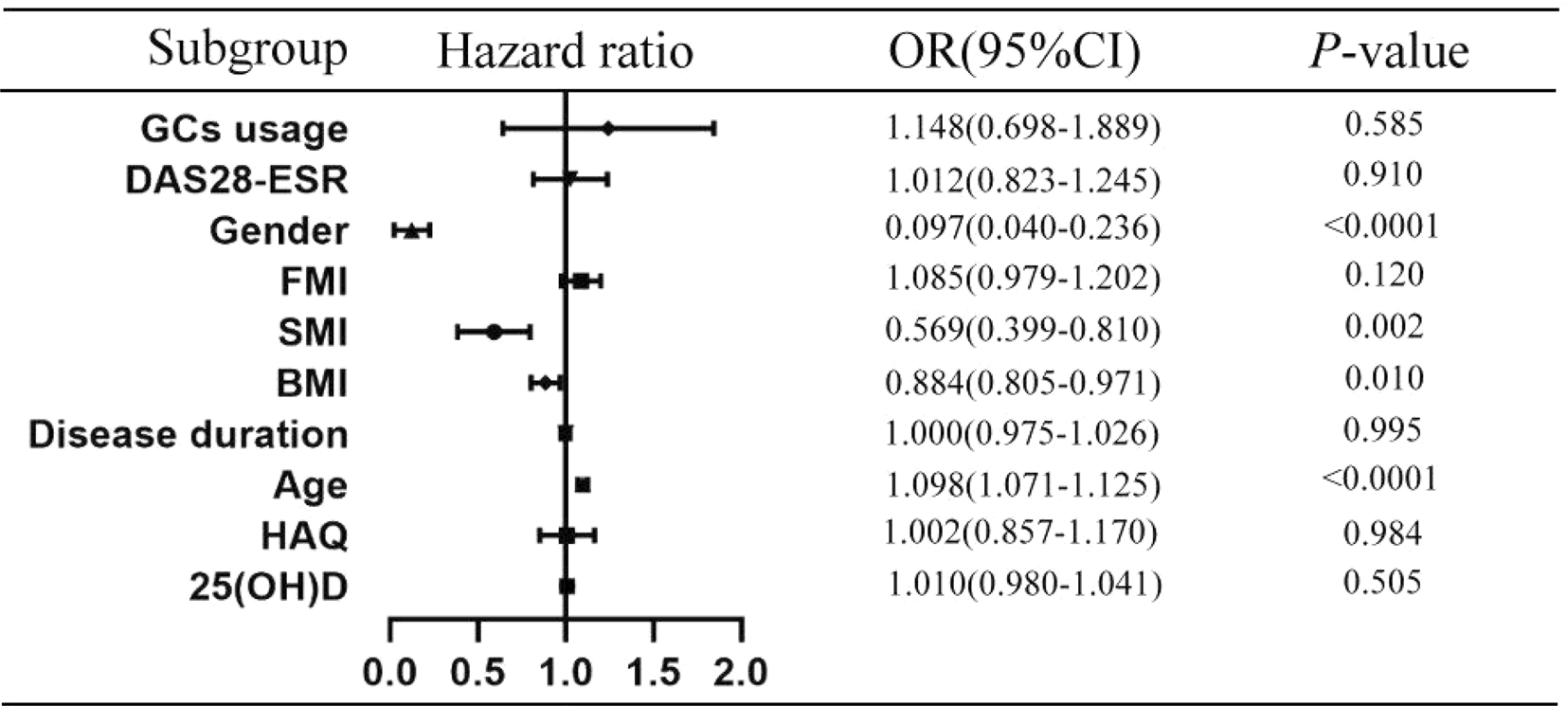
Logistic regression analysis of OP risk factors in RA patients

The linear correlation analysis showed that the SMI in RA patients was positively and linearly correlated with BMD, BMI and FMI at all sites (p < 0. 0001) but negatively correlated with disease duration (p = 0.048), while there was no significant correlation with DAS28-ESR, 25 (OH)D, HAQ and body fat percentage (p = 0.625, p = 0.089, p = 0.393, p = 0.059) (Table 5).The multiple logistic regression analysis was performed with gender (1 = female, 2 = male), age, GC usage, DAS28-ESR, disease duration, BMI, SMI, FMI, 25 (OH)D and HAQ as the independent variables and whether osteoporosis occurred (0 = no, 1 = yes) as the dependent variables. The results showed that SMI (OR = 0.569, p = 0.002, 95% CI 0.399–0.810), BMI (OR = 0.884, p = 0.010, 95% CI 0.805–0.971) and gender (OR = 0.097, p < 0.0001, 95% CI 0.040–0.236) were protective factors for osteoporosis in RA patients, while age (OR = 1.098, p < 0.0001, 95% CI 1.071–1.125) was a risk factor for osteoporosis in RA patients (Fig. 3).

**Table 5:** Correlation between SMI and other indicators in RA patients

Indicators	SMI		Indicators	SMI	
	r	*p*		r	*p*
Neck BMD (g/m^2^)	0.249	< 0.0001	L4 BMD (g/m^2^)	0.252	< 0.0001
Hip BMD (g/m^2^)	0.276	< 0.0001	FMI (g/m^2^)	0.796	< 0.0001
L1 BMD (g/m^2^)	0.205	< 0.0001	Body fat percentage (%)	− 0.090	0.059
L2 BMD (g/m^2^)	0.205	< 0.0001	Disease duration (year)	− 0.094	0.048
L3 BMD (g/m^2^)	0.240	< 0.0001	DAS28	− 0.023	0.625
25 (OH)D (ng/ml)	0.081	0.089	HAQ	− 0.041	0.393

## Discussion

RA is a systemic inflammatory autoimmune disease characterized by chronic synovitis and joint structural destruction. A large number of studies have shown that its mechanism is the activation of macrophages in the synovium, which produces a large number of inflammatory cytokines, such as tumor necrosis factor α (TNF-α), interleukin 1 (IL-1), interleukin 6 (IL-6) and interleukin 8 (IL-8) [17, 18], which promotes the synovium in a chronic inflammatory state, resulting in synovitis and the formation of invasive pannus. It can further cause cartilage and bone destruction and eventually cause serious joint deformity and even disability [19]. Osteoporosis is a systemic bone disease characterized by decreased bone mass and/or degeneration of bone microstructure, resulting in decreased bone strength and increased fragility, which is prone to fracture. The prevalence of osteoporosis in RA is approximately two times that in normal people [20]. The BMDs of Neck, Hip and L1-4 in the RA patients were significantly lower than those in healthy controls. The prevalence of osteoporosis was approximately three times that of the health control group, which was slightly higher than that of other studies at home and abroad. This may be related to the fact that there were more postmenopausal women (male: female = 1:4) in this study. The risk of osteoporosis in postmenopausal women was significantly higher than that in men, making the prevalence of osteoporosis generally higher than that in previous studies.

BMI is a comprehensive reflection of people’s physical fitness, and it is currently recognized as an important indicator affecting the occurrence of BMD and osteoporosis [21, 22]. Most scholars believe that a low BMI is a risk factor for the occurrence of osteoporosis [23, 24], and some studies believe that increasing body weight can help strengthen bone strength, delay the occurrence of osteoporosis and reduce the degree of osteoporosis [25]. A randomized trial of alendronate for osteoporosis prevention found that low BMI was considered a risk factor for low BMD or increased bone loss; women with the lowest body fat percentage or BMI had 12% lower BMD at baseline and more than doubled two year bone loss compared with women with the highest body mass index [26]. Kweon et al. [27] showed that BMI ≤ 22 kg/m^2^ was an independent risk factor for osteoporosis at any site in male RA patients (OR = 3.43, p = 0.043). Some scholars believe that both a low BMI and high BMI are risk factors for osteoporosis; therefore, the relationship between BMI and osteoporosis still needs to be further studied and explored. Since most studies have considered a low BMI as a risk factor for osteoporosis, many people believe that thinner people are prone to osteoporosis, while obesity is not. However, underweight or obesity according to BMI is actually not able to identify the role of fat or muscle in BMI. SMI and FMI (reflecting muscle and fat, respectively) are important components of BMI. Is there a close relationship between the significantly increased prevalence of osteoporosis in RA patients and SMI or FMI? Dogan et al. [28] included 30 female RA patients and 30 female controls. The results showed that the SMI of RA patients was significantly lower than that of the control group (5.83 ± 0.80 vs. 7.30 ± 1.64, p = 0.022), and the overall prevalence of sarcopenia in RA patients was 43.3%, 4.3 times that of the health control group (10.0%) (χ^2^ = 8.52, p = 0.004). In our previous study [29], we determined the skeletal muscle quality of 188 RA patients and also found that the prevalence of sarcopenia in patients with RA was 63.8%; in the control group it was only 9.0% (χ^2^ = 107.884, p < 0.0001), which was approximately seven times that of the healthy control group, and the result was slightly higher than that in foreign literature. This study again found that the SMI in the RA patients was significantly lower than that in the controls, and the prevalence of sarcopenia in the RA patients was 54.5%, which was approximately six times higher than that in the healthy controls (9.0%) (χ^2^ = 96.747, p < 0.0001), similar to the results of the above studies. Lim et al. [30] included 1767 Korean premenopausal women, and the results showed that the prevalence of low BMD and low SMI in low-weight (BMI < 19.5 kg/m^2^) women was 23.9% and 18.4%, respectively, which was significantly higher than the prevalence of 9.4% and 1.7% in normal-weight women. The adjusted results show that compared with normal-weight women, underweight women had a higher risk of low BMD (OR = 3.41, 95% CI 2.31–5.05), low SMI (OR = 11.61, 95% CI 6.17–21.88) and both low BMD and low SMI (OR = 23.82, 95% CI 8.92–63.58), suggesting an inevitable association between BMI and BMD and SMI. Almeida et al. [31] also found that BMI and total hip BMD in patients with sarcopenia were significantly lower than those without sarcopenia (p < 0.0001, p = 0.004). Dogan et al. [28] also found that among female RA patients, female patients with normal BMI or overweight were more likely to have sarcopenia than obese patients (46.2% vs. 7.6%, χ^2^ = 15.13, p = 0.001), which suggests that there are certain defects in judging obesity based on BMI alone, and the influence of muscle needs to be taken into account. The results of this study showed that SMI increased with increasing BMI in RA patients, and SMI was positively correlated with BMI (p < 0.0001), suggesting that increased BMI may be accompanied by increased muscle mass. Therefore, SMI was decreased, and the prevalence of sarcopenia was significantly increased in RA patients. BMI is a protective factor for osteoporosis in RA to a certain extent, but due to the positive correlation between SMI and BMI, it is necessary to further consider that there may be a close relationship between SMI and secondary osteoporosis in RA patients.

Verschueren et al. [32] studied the correlation between sarcopenia and BMD in 679 elderly men. The results showed that appendicular skeletal muscle, fat content and muscle strength were linearly correlated with BMD in various parts. Ning et al. [33] found a negative linear relationship between sarcopenia and regional/total body BMD in non-Hispanic white, non-Hispanic black and non-Hispanic Asian men (p < 0.05). He et al. [34] showed that the risk of osteopenia/osteoporosis increased 1.8 times in sarcopenia patients compared with normal people. Baker et al. [35] enrolled 112 RA patients (55 men) and 412 controls (194 men), and they found that RA patients had higher BMI Z scores (p < 0.001), lower appendicular lean mass index (ALMI) Z scores after adjusting for FMI (p = 0.02), lower muscle strength Z scores (p = 0.01) and lower muscle density Z scores (p < 0.001) compared with the controls. The ALMI Z score of RA patients was positively correlated with BMD (β: 0.29 (0.062–0.52); p = 0.01). The results of this study also showed that SMI was positively correlated with BMD at all sites in RA patients (p < 0.0001), suggesting a close relationship between SMI and osteoporosis in RA. However, when BMI was fixed, especially when BMI was normal or overweight, the prevalence of osteoporosis in the sarcopenia group was significantly higher than that in nonsarcopenia group (p = 0.001, p < 0.0001). Under a fixed SMI, the prevalence of osteoporosis in the RA patients without sarcopenia was different among the different BMI groups (66.7%, 21.7% and 13.0%, p = 0.039), while in the RA patients with sarcopenia, there was no significant difference in the prevalence of osteoporosis among the RA patients with different BMIs (44.4%, 42.8% and 61.1%, p = 0.128). This suggests that the intrinsic association between muscle and osteoporosis may be the essential factor in the apparent correlation between BMI and osteoporosis and that SMI is the main factor affecting the occurrence of osteoporosis in RA patients. Mechanistically, the increased secretion of TVF-α, IL-1β, IL-6, INF-γ and cyclooxygenase-2 (COX-2) in RA patients may not only cause synovitis but also activate nuclear factor κB and ubiquitin-protein bypass, leading to increased protein degradation in muscle. Some studies have found that muscle cytokines, such as IL-6 and TNF-α myostatin, also play an important role in the decomposition of the musculoskeletal system [36]. In addition, the increased expression of the COX-2 gene in RA patients can inhibit the production of insulin-like growth factor-1 (IFG-1) and cause muscle loss [37] as well as malnutrition in RA patients, joint function damage leading to reduced exercise and long-term use of drugs can lead to muscle loss. Approximately 66% of RA patients had a decrease in the number of skeletal muscle cells, consumption of muscle protein and loss of function [38]. Therefore, there is a general reduction in the muscle mass in RA patients, and this reduction in muscle mass may be inseparable from the secondary osteoporosis in RA patients. There are also some limitations in our study. First, the RA patients we evaluated were all hospitalized patients from a single hospital, which may have led to selection bias. Second, the patients with RA outnumbered the normal controls, which would lead to overestimation of the results derived from our data. Third, this study enrolled far more women than men; thus, the conclusions may not be appropriate for men with RA, and further studies with a larger number of male patients are needed. Nevertheless, this study is meaningful and uncommon for the exploration of the association between BMI, SMI and FMI with secondary osteoporosis in RA.

## Conclusions

In conclusion, although BMI is associated with the prevalence of osteoporosis in RA patients, and the prevalence of osteoporosis is inseparable from the increase in age and use of hormones, SMI is the most important protective factor affecting secondary osteoporosis in RA. RA patients often have osteoporosis and have a higher prevalence of sarcopenia. Therefore, attention should be paid to the influence of muscle on RA and its secondary osteoporosis. In the process of diagnosis and treatment, both muscle and sarcopenia must be taken into account so as to minimize the prevalence of osteoporosis in RA patients.

## Data Availability

All data generated or analysed during this study were included in this published article.

## Code availability

All data analysis was carried out using Statistical Package for Social Sciences (SPSS) v.17.0
